# Seasonal differences in depressive affect, worry-related sleep disturbance, and low energy among UK young people

**DOI:** 10.1371/journal.pone.0356293

**Published:** 2026-08-14

**Authors:** Xu Guan, Qi Ding, Yanzhi Li

**Affiliations:** 1 School of Health in Social Science, The University of Edinburgh, Edinburgh, United Kingdom; 2 Florence Nightingale Faculty of Nursing, Midwifery & Palliative Care, King’s College London, London, United Kingdom; SMS Medical College and Hospital, INDIA

## Abstract

Seasonal differences in young people’s mental health may be concentrated in specific symptoms. This population-based repeated cross-sectional study examined winter–non-winter differences in depressive affect, worry-related sleep disturbance and low energy among people aged 16–24 years in the United Kingdom. Understanding Society Calendar Year Datasets were analysed for 2022–2023, with extensions covering 2020–2023. Winter was defined as an interview data in December, January or February. The primary outcome was the mean of three equally weighted standardised symptom components. Standardised Pearson alpha was 0.717 and ordinal alpha was 0.737, supporting use of the index as a shared descriptive measure alongside component-level analysis. Targeted maximum likelihood estimation with Super Learner estimated covariate-standardised mean contrasts. Survey weights informed point estimation and respondent clustering informed variance estimation. Full survey-design regression and inverse-probability-of-complete-case weighting provided complementary checks. The main analysis included 5,949 person-interview observations from 4,167 young people. The winter–non-winter difference was 0.081 index points, with a 95% confidence interval from 0.029 to 0.132. On the original scales, low energy differed by 0.119 points on a 0–4 scale and depressive affect by 0.080 points on a 0–4 scale. Worry-related sleep disturbance differed by 0.044 points on a 0–3 scale, with greater uncertainty. Estimates were similar across analytical approaches and calendar periods, while winter remained at the upper end of the adjusted four-season profile. This period is for closer youth mental health monitoring and for making existing low-intensity support more visible and accessible.

## Introduction

Depressive symptoms and mental wellbeing may vary across seasons, and winter is a plausible period of higher depressive symptom burden in population-based mental health research. Young people’s mental health may be sensitive to seasonal variation. Large-scale evidence from repeated observations indicates that mental health and wellbeing are not evenly distributed across the year, with poorer average mental health indicators in winter than in summer [1]. Population-based evidence also suggests that depressive symptoms may show seasonal patterning at symptom level, particularly among younger respondents [2]. Yet the evidence base remains heterogeneous, with variation in study design, symptom measurement and seasonal definitions [3]. It remains unclear how large any winter–non-winter difference is among UK young people and whether it is concentrated in mood, sleep-related distress or energy.

Young people aged 16–24 form a relevant population for studying seasonal symptom differences because sleep timing, daily routines and social roles are still changing during this life stage. Late adolescence and early adulthood are associated with changes in sleep-wake timing, including a shift in chronotype around the end of adolescence [4]. Cognitive and affective development continues during this period, alongside a strong developmental role for peer relationships [5,6]. At the same time, late adolescence and early adulthood also include transitions in education, employment, independent living and social roles [7]. Long-term increases in psychological distress among young adults in Great Britain add to the public health relevance of understanding contextual influences on symptoms in this age group [8]. Seasonal changes in light exposure, routines and social participation may therefore be reflected differently across mood, sleep-related and energy symptoms.

This study is informed by seasonal affective disorder and seasonality research, which suggests that depressive states may vary with seasonal context. Shorter daylight and shifts in light–dark timing may affect circadian and sleep–wake regulation linked to alertness, energy and mood [9]. Evidence from major depressive disorder admissions also suggests that depressive seasonality may be observed outside narrowly defined seasonal affective disorder [10]. The Dual Vulnerability Hypothesis distinguishes vulnerability to depression from vulnerability to seasonal change, offering a broad framework for understanding variation in winter responses [11]. Winter may also change physical activity, daily routines and face-to-face social contact, which are relevant to psychological distress in young people [6,12]. Seasonal infections and reduced activity may contribute to physical fatigue and low energy. Season therefore represents a combined seasonal context that may include light, weather, infection, academic schedules, holidays, activity and social contact.

Seasonal differences may not be expressed uniformly across individual symptoms. A composite index can summarise the shared direction across related symptoms, while component analyses are needed to identify which symptoms contribute most clearly to the overall contrast. Depressive affect, worry-related sleep disturbance and low energy capture related affective, sleep-related and energy-related experiences that may show different seasonal patterns. Lukmanji et al. found that specific depressive symptoms did not vary uniformly across seasons and that symptom-level seasonality was more extensive among younger respondents [2].

The 2020–2023 observation window also spans markedly different social and public-health conditions. During this period, restrictions, infection levels, education, work and social contact changed repeatedly [13]. These changes are relevant to the social side of seasonal vulnerability because winter may be experienced differently when everyday routines, institutional schedules and social participation are disrupted [12]. Trends in young adult mental health during these years also occurred within longer-term changes that began before the pandemic [8,14]. A descriptive comparison of earlier and later calendar periods can show whether the winter contrast was concentrated in one part of the observation window.

Population-based UK evidence remains limited on the magnitude, symptom composition and temporal consistency of winter–non-winter differences among young people [3]. Using Understanding Society Calendar Year Datasets, we conducted a population-based repeated cross-sectional analysis of young people aged 16–24 years. The main analysis used 2022–2023 data, with extensions covering 2020–2023 and comparing the earlier 2020–2021 and later 2022–2023 calendar periods. We also estimated covariate-standardised winter–non-winter mean differences in the composite symptom index and its three components. We hypothesised that winter assessment would be associated with a higher mean composite symptom index than non-winter assessment, and examined the original-scale component differences, internal consistency of the index, calendar-period variation and a four-season definition of assessment season.

## Materials and methods

### Study design, data source and ethics

This study used a pooled repeated cross-sectional design to estimate winter–non-winter differences in a composite symptom index and its component symptoms in population data. We used person-interview observations from adult individual interview files in the Understanding Society Calendar Year Datasets, 2020–2023, which support calendar-year cross-sectional analysis of individuals and households [15–18]. The unit of analysis was the person-interview observation.

The primary analysis used the 2022–2023 calendar-year data. The extension analyses pooled observations from 2020 to 2023 and compared estimates for the earlier calendar period, 2020–2021, with those for the later calendar period, 2022–2023. Participants could contribute observations in more than one calendar year. All eligible annual person-interview observations were retained, including multiple observations contributed by the same respondent.

Understanding Society obtained ethical approval for the relevant main-study waves through the University of Essex Ethics Committee. Waves 10 and 11 were covered by the Committee’s approval letter dated 4 October 2016. Waves 12–15 were approved under references ETH1920−0123, ETH2021−0015, ETH2122−0246 and ETH2223−0264, respectively. Participants completing face-to-face or telephone interviews gave oral informed consent. Participants completing web interviews provided electronic informed consent. The adult individual questionnaire is administered from age 16 years. This study was a secondary analysis of de-identified data accessed through the UK Data Service End User Licence and involved no direct contact with participants. The data were accessed for research purposes on 24 April 2026.

### Participants

The analytical population was UK young people aged 16–24 years with adult interview data. Eligible observations were completed adult individual interviews from respondents aged 16–24 years with a valid recorded interview month. Survey-weighted analyses additionally required a valid positive calendar-year adult main-interview survey weight. The adult main-interview survey weight is the cross-sectional survey weight supplied for adult individual interviews. It accounts for unequal selection probabilities and differential interview response, allowing weighted estimates to represent the survey target population. Observations without a valid positive weight could not make a defined contribution to the survey-weighted estimand. The primary complete-case analysis further required complete data for all three outcome components and all prespecified adjustment variables. S1 Table reports sample formation, complete-case retention by season, and comparisons between included and excluded observations.

### Winter assessment

The exposure was winter season at assessment. Observations with a recorded interview start month in December, January or February were classified as winter, and observations in all other months were classified as non-winter. The recorded interview month was used to operationalise the seasonal exposure. This definition followed the UK Met Office convention that meteorological winter runs from 1 December to the final day of February [19].

### Outcomes

The primary outcome was a three-component symptom index comprising depressive affect, worry-related sleep disturbance, and low energy. These dimensions were selected a priori to capture affective and vegetative aspects of depressive symptom burden, consistent with psychiatric classifications that include low mood, sleep disturbance and reduced energy or fatigue within depressive episodes [20,21]. The components were combined into a symptom pattern, while also being analysed separately because seasonal variation may differ across depressive symptoms in young people [2]. Depressive affect and low energy were derived from SF-12 items, and worry-related sleep disturbance was derived from a General Health Questionnaire item. The exact item wording, response options, raw coding, and recoding are reported in S2 Table.

Depressive affect was reverse-coded to a 0–4 scale, with higher values indicating more frequent depressed affect. Worry-related sleep disturbance was coded from 0 to 3, with higher values indicating greater sleep loss over worry. Low energy was reverse-coded to a 0–4 scale, with higher values indicating lower energy. Negative survey missing-value codes were treated as missing.

Each component was standardised using its survey-weighted mean and standard deviation in the 2022–2023 outcome-complete reference sample. The composite symptom index was the arithmetic mean of the three standardised component scores. This approach gave each component equal weight despite their different original score ranges. The composite was not re-standardised after averaging. Estimates are therefore reported as points on the composite symptom index, defined as the mean of the component z scores. For auxiliary standardised interpretation, the primary estimate was also divided by the survey-weighted standard deviation of the composite symptom index and expressed in units of its own distribution. The three component outcomes were also analysed separately on their recoded original scales to make the magnitude of the winter–non-winter contrasts more interpretable. Component contrasts were additionally expressed as percentages of the obtainable score range for descriptive interpretation.

Internal consistency of the composite symptom index was evaluated using standardised Pearson alpha, ordinal alpha, pairwise component correlations, and corrected item-rest correlations. Confidence intervals were estimated from 1,000 bootstrap resamples clustered by respondent identifier. These analyses assessed internal consistency. We also repeated the primary analysis using a two-component index comprising depressive affect and low energy, excluding worry-related sleep disturbance. This sensitivity analysis is reported in S4 Table.

### Adjustment variables

The adjustment strategy compared winter and non-winter observations under a common observed covariate distribution. Age and sex represented demographic composition. Calendar year and survey wave represented temporal and fieldwork structure. Grouped economic activity and educational attainment represented social and educational position. Long-standing health condition and self-rated general health represented observed health status. UK region and urban or rural residence represented geographical context. The set was fixed before outcome modelling, without automated or significance-based selection. This approach addresses measured compositional differences between winter and non-winter assessments. Residual confounding from seasonal factors not captured in the data, including daylight, weather, infections, academic schedules, holidays, social activity, and seasonal survey participation, remains possible [22].

### Survey weights and design variables

The survey design was handled separately for population representation and variance estimation. The adult main-interview survey weight was used to calculate survey-weighted descriptive estimates and to define the weighted target population for point estimation. The weight was used for survey-weighted descriptive estimates and point estimation in the primary targeted maximum likelihood estimation (TMLE) analysis. It also served as the analysis weight in the full-design survey-weighted regression benchmark and as the base survey weight in the inverse-probability-of-complete-case weighting (IPCCW) sensitivity analysis.

Pooling calendar years required a common population scale and calendar-year-specific design identifiers. Each year-specific adult main-interview weight was divided by the number of included calendar years. This scaling defined the pooled target as an average calendar-year population. Stratum and primary sampling unit identifiers were made calendar-year specific before pooling. The weights, strata, and primary sampling units were jointly incorporated in descriptive analyses and the full-design survey-weighted regression benchmark [23]. The strata and primary sampling units also defined the resampling scheme for the survey-design bootstrap used in the IPCCW sensitivity analysis [24].

For the TMLE, empirical-influence-curve standard errors were clustered by respondent identifier to allow for repeated interviews from the same individual [25]. Published strata and primary sampling units were not included in the TMLE variance calculation. The corresponding confidence intervals therefore account for within-respondent dependence but are not full complex-survey variance estimates. Full design-based inference was evaluated separately through the survey-weighted regression benchmark and the survey-design bootstrap IPCCW sensitivity analysis.

### Estimand

The estimand was the covariate-standardised mean difference between winter and non-winter assessments over the observed covariate distribution:


$$\begin{array}{c} \psi =E_{W}\left\{E\left(Y|A=\text{1},W\right)-E\left(Y|A=\text{0},W\right)\right\}. \end{array}$$

Let A denote the winter season, Y denote the outcome and W denote the adjustment variables. This contrast compares the mean composite symptom index or component score under winter and other seasons over the observed covariate distribution. Because winter exposure was observational, estimates were interpreted as covariate-standardised winter–non-winter contrasts, contingent on exchangeability, positivity, consistency and measurement assumptions [26,27].

### Statistical analysis

Analyses began with unweighted sample-flow counts and survey-weighted characteristics. Table 1 reports weighted summaries with unweighted observation counts. Seasonal complete-case retention and included-versus-excluded comparisons are reported in S1 Table.

**Table 1:** Survey-weighted baseline characteristics of the 2022–2023 analytic sample.

Characteristic	Non-winter interviews (n = 4,493)	Winter interviews (n = 1,456)
Person-interview observations	4,493	1,456
Age, years	19.93 (2.60)	20.10 (2.57)
**Sex**		
Female	2,490 (49.6)	802 (47.1)
Male	2,003 (50.4)	654 (52.9)
**Calendar year**		
2022	1,975 (45.2)	623 (44.6)
2023	2,518 (54.8)	833 (55.4)
**Survey wave**		
Wave 12	4 (0.2)	15 (1.5)
Wave 13	869 (21.8)	266 (21.0)
Wave 14	2,362 (53.1)	712 (49.5)
Wave 15	1,258 (24.8)	463 (28.0)
**Economic activity**		
Employed or apprentice	2,035 (45.0)	630 (42.7)
Full-time student	1,846 (38.9)	650 (43.8)
Unemployed	423 (10.8)	116 (9.3)
Long-term sick or disabled	49 (1.6)	23 (2.1)
Other inactive or other	140 (3.6)	37 (2.2)
**Educational attainment**		
Degree	695 (14.4)	237 (14.7)
Other higher qualification	247 (4.9)	69 (4.6)
A-level or equivalent	1,716 (36.6)	647 (43.8)
GCSE or equivalent	1,440 (34.2)	408 (29.7)
Other qualification	72 (2.0)	17 (1.2)
No qualification	323 (7.9)	78 (5.9)
**Long-standing health condition**		
Yes	839 (19.3)	296 (22.8)
No	3,654 (80.7)	1,160 (77.2)
**Self-rated general health**		
Excellent	793 (18.0)	276 (19.2)
Very good	1,656 (36.2)	549 (35.3)
Good	1,431 (31.6)	455 (31.8)
Fair	515 (11.5)	140 (10.0)
Poor	98 (2.7)	36 (3.6)
**UK region**		
North East	157 (3.7)	55 (4.5)
North West	495 (11.9)	178 (12.3)
Yorkshire and the Humber	422 (7.8)	154 (8.6)
East Midlands	287 (6.6)	104 (7.4)
West Midlands	468 (9.9)	107 (5.8)
East of England	378 (8.6)	127 (10.4)
London	597 (15.2)	164 (11.9)
South East	505 (13.1)	175 (14.3)
South West	376 (9.1)	112 (7.3)
Wales	213 (4.0)	55 (4.3)
Scotland	338 (7.7)	131 (9.9)
Northern Ireland	257 (2.4)	94 (3.2)
**Urban-rural residence**		
Urban	3,550 (80.7)	1,146 (78.6)
Rural	943 (19.3)	310 (21.4)

The primary analysis used targeted maximum likelihood estimation to estimate 2022–2023 winter–non-winter contrasts for the composite symptom index and its three components. Super Learner was used to reduce reliance on a single prespecified functional form by combining conventional regression and flexible algorithms through cross-validation [28,29]. Ten-fold cross-validation was grouped by respondent. Outcomes were mapped to the unit interval for logistic targeting and returned to their analysis scales. Predicted winter probabilities were bounded at 0.025 and 0.975. Empirical influence-curve standard errors were clustered by respondent, with two-sided 95% confidence intervals.

**Fig 1:**
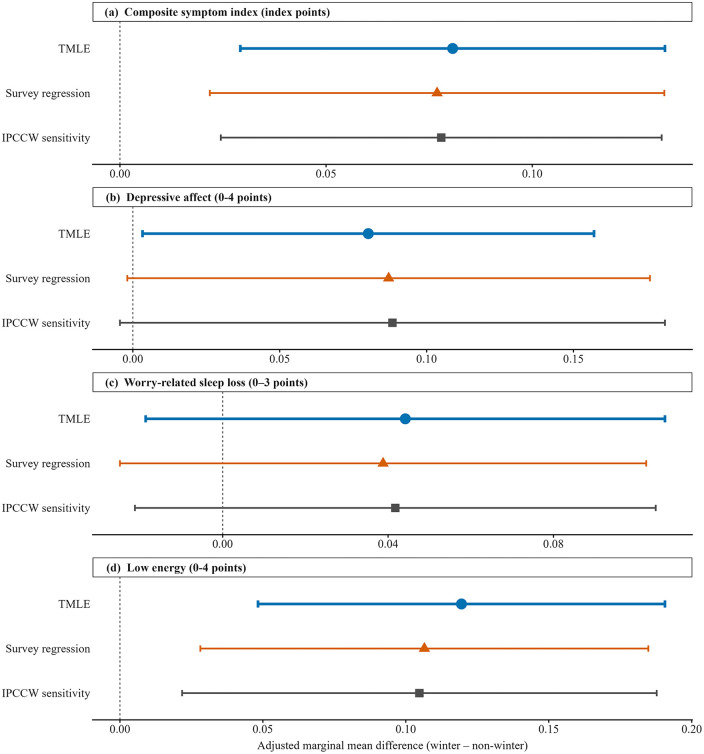
Agreement of winter–non-winter estimates across analytic approaches. ^a^ Positive values indicate higher symptom burden in winter. The composite index is expressed in SD units; component rows retain their original recoded score scales.

To provide an interpretable conventional comparison, method agreement was assessed using Gaussian identity-link survey-weighted regression models for the four outcomes. These models used the same complete cases and adjustment variables while incorporating weights, strata, and primary sampling units. Winter coefficients were compared descriptively with TMLE estimates in Fig 1 and S3 Table.

Complete-case selection was examined through unweighted and survey-weighted included-versus-excluded comparisons. Stabilised inverse-probability-of-complete-case weighting was then applied within the positive-weight eligible sample [30]. The completion model included winter, year, grouped wave, and natural-spline terms for age and log base survey weight. The untruncated multiplier was primary, with 1st and 99th percentile truncation as a tail-sensitivity analysis. Combined weights were applied to the full-design regression models. Confidence intervals used 500 full-process Rao–Wu rescaled primary-sampling-unit bootstrap replicates that refitted the completion and outcome models [24,31].

Temporal analyses repeated the composite TMLE in pooled 2020–2023 data and in the earlier 2020–2021 and later 2022–2023 periods. A direct earlier-minus-later difference compared the period-specific contrasts.

A four-season survey-weighted regression examined dependence on the binary winter definition. Adjusted seasonal marginal means and an overall 3-df Wald test are reported in S1 Fig. Survey wave was omitted because structural season-by-year-by-wave zero cells would require unsupported extrapolation.

Implementation checks removed the interaction regression learner, used the two-component index, summarised predicted winter probabilities, and verified respondent-grouped fold integrity. These outcome-construction and implementation sensitivities are reported in S4 Table. Analyses used R version 4.6.0.

## Results

### Sample formation and missing-data selection

The analytic sample preserved broad coverage of eligible interviews and supported population-weighted comparison across seasons. Of 6,675 age and month eligible person-interviews, 6,320 had a finite positive adult main-interview weight and 5,949 entered the complete-case analysis. The final sample represented 4,167 participants, including 4,493 non-winter and 1,456 winter assessments. Complete cases retention was high in both groups, reaching 88.6% outside winter and 90.7% in winter. On the survey-weighted scale, winter retention exceeded non-winter retention by 2.3 percentage points (95% CI 0.1 to 4.6, p = 0.044). S1 Table shows the sample pathway and identifies the observed characteristics contributing to selection. Table 1 describes the weighted analytic population.

The winter and non-winter groups were broadly comparable, although small compositional differences supported covariate-standardised estimation. Table 1 shows that weighted mean age was similar in non-winter and winter interviews, at 19.93 and 20.10 years. The sex distribution also differed little across interview seasons. Winter assessments included more full-time students and respondents with a long-standing health condition, alongside a smaller urban proportion. Educational composition varied modestly, with winter interviews including a higher weighted proportion of respondents reporting A-level or equivalent qualifications and a lower proportion reporting GCSE or equivalent qualifications.

### Measurement evidence for the composite symptom index

The composite symptom index captured a coherent symptom pattern while preserving information from distinct domains. Standardised Pearson alpha was 0.717 (95% CI 0.687 to 0.732), with ordinal alpha of 0.737 (95% CI 0.713 to 0.761). Pairwise correlations ranged from 0.419 to 0.485, describing interrelatedness among the components. Corrected item-rest correlations ranged from 0.516 to 0.567, so every component contributed positively to the common score. Together, these results support the internal consistency of the index as a dimensional measure of depressive affect, worry-related sleep disturbance, and low energy. S2 Table links this evidence to the original item wording, response scales, recoding and component distributions.

### Primary effect and practical scale

The winter contrast was expressed across all three symptom domains, with the clearest component-level difference in low energy. The TMLE estimate was 0.081 points on the composite symptom index (95% CI 0.029 to 0.132), indicating a higher adjusted winter mean. As an auxiliary standardised interpretation, this estimate corresponded to 0.103 of the survey-weighted SD of the composite symptom index (95% CI 0.037 to 0.169). Original-score estimates made the component pattern more tangible. Low energy differed by 0.119 points on its 0–4 scale (95% CI 0.048 to 0.191), equivalent to 3.0% of the obtainable range. Depressive affect differed by 0.080 points on its 0–4 scale (95% CI 0.003 to 0.157), equivalent to 2.0% of the range. Worry-related sleep disturbance differed by 0.044 points on its 0–3 scale (95% CI −0.019 to 0.107), equivalent to 1.5% of the range. Fig 1 presents the comparative effect profile, with exact estimates in S3 Table.

### Convergence across analytical approaches

The strongest analytical feature was the close agreement between estimators with different modelling and variance structures. The TMLE estimate of 0.081 was closely matched by the full-design survey-weighted regression estimate of 0.077 (95% CI 0.022 to 0.132). IPCCW produced an estimate of 0.078 (95% CI 0.024 to 0.131) after reweighting complete cases towards the positive-weight eligible population. Across all four outcomes, full-design regression and IPCCW preserved the direction and ordering of the primary estimates. Low energy remained the largest component contrast, followed by depressive affect and worry-related sleep disturbance. IPCCW changed the corresponding complete-case regression estimates by no more than 0.003 points. Truncating the completion multiplier at its 1st and 99th percentiles changed estimates by no more than 0.0012 points. The central effect profile was therefore maintained under flexible TMLE, conventional full-design regression, and measured-selection weighting. Fig 1 provides the visual comparison, and S3 Table reports the exact intervals.

### Calendar-period extension

**Fig 2:**
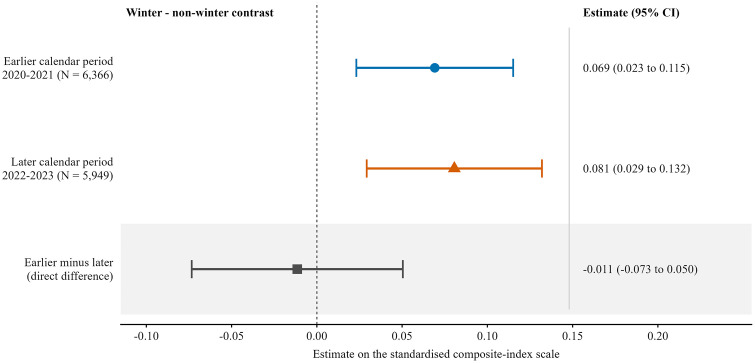
Calendar-period comparison of winter-non-winter composite-index contrasts. ^b^ Positive period-specific values indicate higher mean composite symptom burden in winter. The direct difference is defined as the earlier (2020-2021) estimate minus the later (2022-2023) estimate; positive values would indicate a larger contrast in the earlier period.

The winter contrast was observed across the full analysis window and in both calendar blocks. The pooled 2020–2023 estimate was 0.073 index points (95% CI 0.037 to 0.109). Period-specific estimates were 0.069 for 2020–2021 (95% CI 0.023 to 0.115) and 0.081 for 2022–2023 (95% CI 0.029 to 0.132). Their direct earlier-minus-later difference was −0.011 (95% CI −0.073 to 0.050). Fig 2 shows the shared direction and between-period uncertainty visible without treating the calendar blocks as measures of restriction exposure. Exact estimates are reported in S4 Table.

### Four seasons sensitivity

The broader seasonal comparison placed winter at the upper end of the adjusted annual profile. The adjusted composite mean was 0.062 in winter (95% CI 0.009 to 0.115). Adjusted means for spring, summer, and autumn clustered between −0.018 and −0.011. The overall four-season test was F(3, 1065) = 2.363, p = 0.070. S1 Fig shows the complete set of adjusted means and intervals. Winter therefore remained the highest adjusted seasonal mean when the binary exposure was replaced by four meteorological seasons.

### Implementation stability

Implementation checks reinforced the stability of the main effect profile. Removing the interaction-augmented regression learner changed reported estimates by no more than 0.0003 points. The two-component symptom index produced a winter contrast of 0.086 (95% CI 0.033 to 0.140), differing by 0.006 points from the primary estimate. Predicted winter probabilities ranged from 0.141 to 0.697. Every respondent remained within one cross-validation fold. The IPCCW model produced completion probabilities from 0.684 to 0.989 and stabilised multipliers no larger than 1.353. All 500 bootstrap replicates completed successfully. These diagnostics and outcome-construction checks are reported in S4 Table.

## Discussion

Winter was associated with a greater likelihood of experiencing symptoms related to mood and energy among UK young people. In England, about one in five people aged 8–25 years had a probable mental disorder in 2023 [32]. Depression and anxiety are also among the leading causes of illness and disability in adolescence, which makes a recurring winter increase in symptom burden relevant to public mental health surveillance [33,34]. The pattern was clearest for low energy and depressive affect, with a smaller positive estimate for worry-related sleep loss. An effect of approximately one tenth of a standard deviation represents a modest shift in the population average, but it identifies a measurable seasonal context shared across UK young people. Small shifts in average mental-health scores can have population relevance when they occur across widely exposed groups and influence the distribution of symptom levels [35].

### Interpretation of the symptom pattern

The composite and component results show a clear symptom-specific pattern. The internal-consistency findings supported the use of the three components as a shared descriptive index, while their correlations indicated that depressive affect, worry-related sleep disturbance, and low energy retained distinct information. This interpretation accords with population evidence that seasonal variation can differ across individual depressive symptoms, particularly among young people [2].

Low energy was the clearest feature of the winter-associated symptom profile. It may be relevant to concentration and sustained engagement, as fatigue has been linked to executive functioning in depression [36]. Low energy is therefore a relevant focus for future studies of concentration, sustained engagement, and participation among young people in education and work.

The sleep result clarifies an important measurement boundary. Worry-related sleep disturbance showed a positive but imprecise contrast, and this differs from winter depression accounts that emphasise hypersomnia, sleep duration or circadian timing [37]. The similar estimate from the two-component index showed that the overall winter contrast was largely maintained when the sleep item was removed. The weaker sleep finding is plausible because the available item measured sleep loss over worry, not seasonal sleep extension or rhythm timing. Taken together, the findings identify a winter-associated mood-and-energy profile with a possible contribution from worry-related sleep loss.

Several interacting biological, behavioural and social processes may contribute to the winter-associated mood-and-energy pattern. The Dual Vulnerability Hypothesis offers a broad framework in which sensitivity to seasonal change may combine with vulnerability to depressive symptoms [11]. Shorter daylight and shifts in light–dark timing may alter circadian and sleep–wake regulation and influence alertness, energy and mood [9]. This pathway may be particularly relevant in late adolescence and early adulthood, when sleep timing is still changing [4]. Winter may also alter physical activity and the regularity of study, work and social routines. Broader disruption to daily routines, especially physical activity and sleep, has been associated with depressive symptoms and psychological distress [12], while reduced social contact may be particularly relevant during adolescence because peer relationships remain developmentally important [6]. CBT-SAD targets reduced activity and negative seasonal beliefs, and trial-based evidence in adults with winter depression links changes in seasonal beliefs to symptom improvement [38,39]. The available sleep item addresses worry-related sleep loss, while these broader pathways require direct measures of sleep timing, duration and light exposure. Future within-person studies should examine these processes together, including physical activity, social contact, infection burden and academic or work schedules.

### Public mental health implications

The concentration of the winter contrast in low energy and depressive affect gives the findings practical value for youth settings. Fatigue and energy loss have been linked to executive functioning in depression [36], making concentration, sustained participation and everyday activity relevant outcomes for seasonal research. Schools, colleges, universities, youth services and primary care can therefore focus local monitoring on fatigue, mood, attendance, participation and help-seeking. English primary-care data show that recorded depression, anxiety and antidepressant prescribing can follow a different seasonal pattern [40]. Linking symptom trends with local service-use data would help settings decide whether existing support should be made more visible or accessible during winter, in line with wider priorities for early and accessible youth mental-health care [41].

The temporal and seasonal sensitivity analyses clarify the conditions under which the primary contrast is most informative. Similar winter contrasts in the earlier and later calendar periods show that the pattern was not confined to one exceptional social context, despite substantial changes in education, social restrictions and daily routines during 2020–2023 [13,14]. This broader temporal range makes season a more credible consideration for repeated youth mental-health monitoring and local planning. The four-season profile further indicates that winter remained at the upper end of the adjusted annual symptom profile, while spring, summer and autumn were more closely grouped. This makes winter a practical contextual factor when repeated youth mental-health estimates are compared across surveys or years with different fieldwork timing.

### Strengths and limitations

The study’s main strength is its ability to describe seasonal differences in current symptoms across a population-based sample of UK young people. The combined use of a composite index and original-scale component outcomes preserved both an overall summary and symptom-level interpretation. This is especially valuable for a seasonal research question because clinical records capture help-seeking and service contact, whereas population surveys can identify smaller shifts in mood and energy across the wider youth population. The repeated calendar-year coverage and alternative seasonal classification further allowed the winter contrast to be examined across different social contexts and under a broader annual profile.

Complementary analytical checks strengthened confidence in the substantive pattern without making the interpretation depend on one modelling approach. The primary TMLE allowed flexible adjustment for observed differences between winter and non-winter interviews, while full-design survey regression provided a conventional comparison and IPCCW examined selection into the complete-case sample.

This study also has some limitations. IPCCW can address selection represented by the completion model, but it cannot recover information related to unmeasured causes of missingness. The primary TMLE variance accounted for repeated observations from the same respondent but did not incorporate the published strata and primary sampling units. Its confidence intervals therefore do not represent full complex-survey variance, although the design-based regression and bootstrap analyses provide direct evidence that the main conclusion was similar under full survey-design inference. The repeated cross-sectional design means that the estimates do not represent within-person seasonal change. Winter was operationalised using recorded assessment month, so the analysis could not separate potential winter-related mechanisms such as daylight, temperature, infection burden, activity, social routines or sleep timing [9,12]. The sleep component also had a specific measurement limitation: it captured worry-related sleep loss, not seasonal sleep duration, hypersomnia or circadian sleep phase. External validity is limited to UK young people aged 16–24 in Understanding Society Calendar Year data, and the meaning of winter may differ in other countries, climates, institutional calendars and age groups. Future studies should combine repeated within-person symptoms with richer environmental, behavioural and social measures. Denser monthly follow-up and linked service-use data would help distinguish environmental pathways, individual seasonal trajectories and population changes with clinical or practical importance.

## Conclusion

During the winter, young people are more likely to experience a combined pattern of low energy, depressive affect and worry-related sleep disturbance in the United Kingdom. The difference was clearest for low energy and depressive affect. Its magnitude is on both the standardised index and the original item scales, so the findings indicate seasonal patterning. Similar estimates across calendar periods and analytical approaches suggest that the pattern was not dependent on a single dataset or model. However, assessment month represents a broad seasonal context and cannot distinguish the contributions of daylight, weather, infections, academic schedules, holidays, social activity or survey participation. The study therefore supports accounting for assessment season when interpreting and comparing youth mental health surveillance data. Repeated within-person research with richer symptom, environmental and behavioural measures is needed to identify the processes underlying these differences and determine whether they influence daily functioning, participation or demand for support.

## Supporting information

S1 Fig Adjusted composite-index means across meteorological seasons. (TIF)

S1 Table Sample formation, complete-case retention, and included-versus-excluded comparisons. (DOCX)

S2 Table Composite symptom-index construction, questionnaire items, internal consistency, and original-scale component contrasts. (DOCX)

S3 Table TMLE, Survey-weighted regression, and IPCCW sensitivity analysis. (DOCX)

S4 Table TMLE extensions and sensitivity analyses. (DOCX)
