# Supplementary material for: Seasonal differences in depressive affect, worry-related sleep disturbance, and low energy among UK young people
Source: PLoS One. 2026 Aug 14;21(8):e0356293. doi: 10.1371/journal.pone.0356293 (PMC13475821; doi:10.1371/journal.pone.0356293)
Supplement: Supplementary file 1 [file pone.0356293.s002.docx]

S1 Table. Sample formation, complete-case retention, and included-versus-excluded comparisons

A. Sample formation

| Eligibility and analysis stage | **Overall, n (%)** | **Non-winter, n (%)** | **Winter, n (%)** |
| --- | --- | --- | --- |
| Age 16–24 with a valid interview month | 6,675 (100.0%) | 5,070 (100.0%) | 1,605 (100.0%) |
| **Finite positive adult main-interview weight** | 6,320 (94.7%) | 4,784 (94.4%) | 1,536 (95.7%) |
| **Complete three primary outcome components** | 6,151 (92.1%) | 4,654 (91.8%) | 1,497 (93.3%) |
| Final analytic complete-case sample | 5,949 (89.1%) | 4,493 (88.6%) | 1,456 (90.7%) |

B. Seasonal complete-case retention and exclusion^[1]^

| **Measure** | **Non-winter** | **Winter** | Winter–non-winter | **P value** |
| --- | --- | --- | --- | --- |
| **Final complete cases, unweighted n (retained from eligible)** | 4,493 (88.6%) | 1,456 (90.7%) | 2.1 percentage points |  |
| **Final complete-case rate, survey-weighted % (95% CI)** | 91.9 (90.4 to 93.5) | 94.3 (92.5 to 96.0) | 2.3 (0.1 to 4.6) | 0.044 |
| **Exclusion rate, survey-weighted % (95% CI)** | 8.1 (6.5 to 9.6) | 5.7 (4.0 to 7.5) | -2.3 (-4.6 to -0.1) | 0.044 |

C. Analytic sample and excluded eligible observations^[2]^

| **Characteristic** | **Level** | Analytic sample, n (unweighted %) | Excluded eligible observations, n (unweighted %) | Analytic sample, survey-weighted % | Excluded eligible observations, survey-weighted % | **Difference, weighted percentage points** |
| --- | --- | --- | --- | --- | --- | --- |
| **Assessment season** | Non-winter | 4,493 (75.5%) | 577 (79.5%) | 75.5% | 81.6% | -6.1 |
|  | Winter | 1,456 (24.5%) | 149 (20.5%) | 24.5% | 18.4% | 6.1 |
| **Calendar year** | 2022 | 2,598 (43.7%) | 501 (69.0%) | 45.1% | 35.1% | 10.0 |
|  | 2023 | 3,351 (56.3%) | 225 (31.0%) | 54.9% | 64.9% | -10.0 |
| **Survey wave** | Wave 12 | 19 (0.3%) | 9 (1.2%) | 0.5% | 0.0% | 0.5 |
|  | Wave 13 | 1,135 (19.1%) | 94 (12.9%) | 21.6% | 22.9% | -1.2 |
|  | Wave 14 | 3,074 (51.7%) | 532 (73.3%) | 52.3% | 59.3% | -7.0 |
|  | Wave 15 | 1,721 (28.9%) | 91 (12.5%) | 25.6% | 17.9% | 7.7 |
| Age, years | 16-19 | 2,633 (44.3%) | 338 (46.6%) | 45.2% | 53.9% | -8.8 |
|  | 20-24 | 3,316 (55.7%) | 388 (53.4%) | 54.8% | 46.1% | 8.8 |
| **Sex** | Female | 3,292 (55.3%) | 409 (56.3%) | 49.0% | 49.8% | -0.8 |
|  | Male | 2,657 (44.7%) | 316 (43.5%) | 51.0% | 49.9% | 1.1 |
|  | Missing/invalid | 0 (0.0%) | 1 (0.1%) | 0.0% | 0.3% | -0.3 |
| **Economic activity** | Employed or apprentice | 2,665 (44.8%) | 286 (39.4%) | 44.4% | 34.7% | 9.7 |
|  | Full-time student | 2,496 (42.0%) | 272 (37.5%) | 40.1% | 35.6% | 4.5 |
|  | Long-term sick or disabled | 72 (1.2%) | 18 (2.5%) | 1.7% | 3.2% | -1.5 |
|  | Missing/invalid | 0 (0.0%) | 43 (5.9%) | 0.0% | 12.1% | -12.1 |
|  | Other inactive or other | 177 (3.0%) | 31 (4.3%) | 3.2% | 3.4% | -0.1 |
|  | Unemployed | 539 (9.1%) | 76 (10.5%) | 10.5% | 11.0% | -0.5 |
| Educational attainment | A-level or equivalent | 2,363 (39.7%) | 199 (27.4%) | 38.4% | 14.3% | 24.1 |
|  | Degree | 932 (15.7%) | 94 (12.9%) | 14.5% | 8.4% | 6.1 |
|  | GCSE or equivalent | 1,848 (31.1%) | 194 (26.7%) | 33.1% | 21.2% | 11.9 |
|  | Missing/invalid | 0 (0.0%) | 162 (22.3%) | 0.0% | 49.1% | -49.1 |
|  | No qualification | 401 (6.7%) | 34 (4.7%) | 7.4% | 4.4% | 3.1 |
|  | Other higher degree | 316 (5.3%) | 30 (4.1%) | 4.8% | 1.2% | 3.6 |
|  | Other qualification | 89 (1.5%) | 13 (1.8%) | 1.8% | 1.5% | 0.3 |
| Long-standing health condition | Missing/invalid | 0 (0.0%) | 72 (9.9%) | 0.0% | 16.7% | -16.7 |
|  | No | 4,814 (80.9%) | 507 (69.8%) | 79.9% | 64.7% | 15.1 |
|  | Yes | 1,135 (19.1%) | 147 (20.2%) | 20.1% | 18.6% | 1.5 |
| **Self-rated general health** | Excellent | 1,069 (18.0%) | 136 (18.7%) | 18.3% | 16.7% | 1.5 |
|  | Fair | 655 (11.0%) | 84 (11.6%) | 11.2% | 10.7% | 0.4 |
|  | Good | 1,886 (31.7%) | 173 (23.8%) | 31.6% | 20.9% | 10.7 |
|  | Missing/invalid | 0 (0.0%) | 112 (15.4%) | 0.0% | 27.0% | -27.0 |
|  | Poor | 134 (2.3%) | 23 (3.2%) | 2.9% | 2.3% | 0.6 |
|  | Very good | 2,205 (37.1%) | 198 (27.3%) | 36.0% | 22.4% | 13.6 |
| Urban or rural residence | Missing/invalid | 0 (0.0%) | 5 (0.7%) | 0.0% | 0.7% | -0.7 |
|  | Rural | 1,253 (21.1%) | 120 (16.5%) | 19.8% | 12.4% | 7.5 |
|  | Urban | 4,696 (78.9%) | 601 (82.8%) | 80.2% | 87.0% | -6.8 |
| UK country of residence | England | 4,861 (81.7%) | 590 (81.3%) | 85.1% | 86.2% | -1.1 |
|  | Missing/invalid | 0 (0.0%) | 5 (0.7%) | 0.0% | 0.7% | -0.7 |
|  | Northern Ireland | 351 (5.9%) | 48 (6.6%) | 2.6% | 3.8% | -1.2 |
|  | Scotland | 469 (7.9%) | 53 (7.3%) | 8.2% | 8.3% | -0.1 |
|  | Wales | 268 (4.5%) | 30 (4.1%) | 4.1% | 1.0% | 3.1 |

1. The unweighted row uses the age- and month-eligible sample. Survey-weighted rows use the positive-weight eligible domain (n = 6,320); the difference is winter–non-winter in percentage points.
2. Unweighted columns compare the final analytic sample (n = 5,949) with all excluded age- and month-eligible observations (n = 726). Survey-weighted columns compare the analytic sample with excluded positive-weight eligible observations (n = 371).
