# Supplementary material for: Seasonal differences in depressive affect, worry-related sleep disturbance, and low energy among UK young people
Source: PLoS One. 2026 Aug 14;21(8):e0356293. doi: 10.1371/journal.pone.0356293 (PMC13475821; doi:10.1371/journal.pone.0356293)
Supplement: Supplementary file 2 [file pone.0356293.s003.docx]

S2 Table. Composite symptom-index construction, questionnaire items, internal consistency, and original-scale component contrasts

A. Questionnaire items and response options

| **Component** | **Variable** | **Original item wording** | **Original response options** |
| --- | --- | --- | --- |
| **Depressive affect** | scsf6c | During the past 4 weeks, how much of the time have you felt downhearted and depressed? | All of the time (1); Most of the time (2); Some of the time (3); A little of the time (4); None of the time (5) |
| **Worry-related sleep disturbance** | scghqb | Have you recently lost much sleep over worry? | Not at all (1); No more than usual (2); Rather more than usual (3); Much more than usual (4) |
| **Low energy** | scsf6b | During the past 4 weeks, how much of the time did you have a lot of energy? | All of the time (1); Most of the time (2); Some of the time (3); A little of the time (4); None of the time (5) |

B. Original coding, survey-weighted standardisation, and composite symptom-index construction

| **Component** | Original coding | **Analysis recoding** | **Final range** | Survey-weighted mean (SD) | Role in composite symptom index |
| --- | --- | --- | --- | --- | --- |
| **Depressive affect** | 1-5; higher raw values indicate less frequent depressive affect | Reverse coded 1-5 to 4-0; higher values indicate more depressive affect | 0-4 | 1.423 (1.121) | Standardised and equally weighted |
| **Worry-related sleep disturbance** | 1-4; increasing response denotes greater sleep loss over worry | Recoded 1-4 to 0-3; higher values indicate more sleep loss over worry | 0-3 | 0.871 (0.824) | Standardised and equally weighted |
| **Low energy** | 1-5; higher raw values indicate more frequent energy | Recoded 1-5 to 0-4; higher values indicate lower energy | 0-4 | 1.761 (1.004) | Standardised and equally weighted |
| Composite symptom index | Not a single original-score scale | Arithmetic mean of the three component z scores; not re-standardised | Index points (mean of component z scores) | Not applicable | Primary three-component symptom index |

C. Internal-consistency evidence for the composite symptom index

| **Statistic** | **Estimate (95% CI)** | **Interpretation** |
| --- | --- | --- |
| **Standardised Pearson alpha** | 0.717 (0.687 to 0.732) | Internal consistency |
| **Ordinal alpha** | 0.737 (0.713 to 0.761) | Ordinal-scale sensitivity analysis |
| **Pearson correlation: depressive affect and sleep disturbance** | 0.485 (0.451 to 0.518) | Positive pairwise association |
| **Pearson correlation: depressive affect and low energy** | 0.470 (0.429 to 0.509) | Positive pairwise association |
| **Pearson correlation: sleep disturbance and low energy** | 0.419 (0.385 to 0.452) | Positive pairwise association |
| Corrected item–rest correlations | 0.516 to 0.567 | All components contribute to the composite symptom index |

D. Original-scale component contrasts from the TMLE

| **Outcome** | **Recoded score scale** | Winter–non-winter contrast (95% CI) | **Share of obtainable score range** |
| --- | --- | --- | --- |
| **Depressive affect** | 0-4 | 0.080 (0.003 to 0.157) | 2.0% |
| **Worry-related sleep disturbance** | 0-3 | 0.044 (-0.019 to 0.107) | 1.5% |
| **Low energy** | 0-4 | 0.119 (0.048 to 0.191) | 3.0% |
