# Supplementary material for: Seasonal differences in depressive affect, worry-related sleep disturbance, and low energy among UK young people
Source: PLoS One. 2026 Aug 14;21(8):e0356293. doi: 10.1371/journal.pone.0356293 (PMC13475821; doi:10.1371/journal.pone.0356293)
Supplement: Supplementary file 3 [file pone.0356293.s004.docx]

S3 Table. TMLE, Survey-weighted regression, and IPCCW sensitivity analysis

| Outcome (analysis scale) | TMLE estimate (95% CI) | Survey-weighted regression estimate (95% CI) | **IPCCW estimate (95% CI)** |
| --- | --- | --- | --- |
| Composite symptom index (index points) | 0.081 (0.029 to 0.132) | 0.077 (0.022 to 0.132) | 0.078 (0.024 to 0.131) |
| **Depressive affect (0-4 points)** | 0.080 (0.003 to 0.157) | 0.087 (-0.002 to 0.176) | 0.088 (-0.004 to 0.181) |
| **Worry-related sleep disturbance (0-3 points)** | 0.044 (-0.019 to 0.107) | 0.039 (-0.025 to 0.102) | 0.042 (-0.021 to 0.105) |
| **Low energy (0-4 points)** | 0.119 (0.048 to 0.191) | 0.106 (0.028 to 0.185) | 0.105 (0.022 to 0.188) |
