# Supplementary material for: Seasonal differences in depressive affect, worry-related sleep disturbance, and low energy among UK young people
Source: PLoS One. 2026 Aug 14;21(8):e0356293. doi: 10.1371/journal.pone.0356293 (PMC13475821; doi:10.1371/journal.pone.0356293)
Supplement: Supplementary file 4 [file pone.0356293.s005.docx]

S4 Table. TMLE extensions and sensitivity analyses

A. Pooled and calendar-period analyses

| Analysis or calendar-period contrast | **Estimate (95% CI)** | **P value** | **Person-interviews** | **Unique participants** |
| --- | --- | --- | --- | --- |
| Primary analysis: 2022–2023 | 0.081 (0.029 to 0.132) | 0.002 | 5,949 | 4,167 |
| Pooled analysis: 2020–2023 | 0.073 (0.037 to 0.109) | <0.001 | 12,315 | 5,928 |
| Earlier calendar period: 2020–2021 | 0.069 (0.023 to 0.115) | 0.003 | 6,366 | 4,125 |
| Later calendar period: 2022–2023 | 0.081 (0.029 to 0.132) | 0.002 | 5,949 | 4,167 |
| Earlier–later direct difference | -0.011 (-0.073 to 0.050) | 0.716 | Not applicable | 5,928 |

B. Model and outcome-construction sensitivities

| **Sensitivity analysis** | Analysis or calendar-period contrast | **Outcome** | **Estimate (95% CI)** | Difference from primary estimate |
| --- | --- | --- | --- | --- |
| Excluding interaction-augmented regression learner | Primary 2022–2023 analysis | Composite symptom index | 0.081 (0.029 to 0.132) | 0.0000 |
| Excluding interaction-augmented regression learner | Primary 2022–2023 analysis | Depressive affect | 0.080 (0.003 to 0.157) | 0.0000 |
| Excluding interaction-augmented regression learner | Primary 2022–2023 analysis | Worry-related sleep disturbance | 0.044 (-0.019 to 0.107) | 0.0000 |
| Excluding interaction-augmented regression learner | Primary 2022–2023 analysis | Low energy | 0.119 (0.048 to 0.191) | 0.0000 |
| Excluding interaction-augmented regression learner | Pooled analysis: 2020–2023 | Composite symptom index | 0.073 (0.037 to 0.109) | -0.0001 |
| Excluding interaction-augmented regression learner | Earlier calendar period: 2020–2021 | Composite symptom index | 0.069 (0.023 to 0.115) | -0.0003 |
| Excluding interaction-augmented regression learner | Later calendar period: 2022–2023 | Composite symptom index | 0.081 (0.029 to 0.132) | 0.0000 |
| Excluding interaction-augmented regression learner | Earlier–later direct difference | Composite symptom index | -0.012 (-0.073 to 0.050) | -0.0003 |
| **Outcome-construction sensitivity** | Primary 2022–2023 analysis | Two-component symptom index excluding worry-related sleep disturbance | 0.086 (0.033 to 0.140) | 0.0057 |
